# A case report and literature review on ovarian lymphangioma

**DOI:** 10.3389/fonc.2024.1476879

**Published:** 2024-10-07

**Authors:** Fang Yang, Wenjing Zhu, Wenjun Shan, Yanping Zhang, Ruilin Li, Wenyan Wang

**Affiliations:** ^1^ Department of Obstetrics and Gynecology, The Second Hospital of Anhui Medical University, Hefei, China; ^2^ Department of Pharmaceutical Sciences, Hefei First People’s Hospital, Hefei, China

**Keywords:** ovarian tumor, lymphangiomas, laparoscopic surgery, case report, immunohistochemistry

## Abstract

Ovarian tumors can be divided into epithelial tumors, germ cell tumors, sex cord-stromal tumors and metastatic tumors according to histological types. Their biological behaviors are different. Lymphangioma is a rare benign tumor that can occur anywhere in the body. Among them, ovarian lymphangioma is particularly rare. The case we reported is the case of ovarian lymphangioma. The patient was admitted to the hospital one month after the physical examination found the ovarian mass. After the examination, the patient was treated with laparoscopic surgery. The patient recovered well after the operation, and no recurrence was found after the follow-up.

## Introduction

Ovarian tumors can be classified based on their tissue origin and pathological characteristics into Functional Cysts, Theca Lutein Cysts, Neoplastic Cysts, etc. (1). Among these, ovarian lymphangioma is a rare benign tumor. Lymphangiomas originate from lymphatic endothelial cells and typically present as a multilocular cystic structure with clear fluid, commonly occurring in subcutaneous tissue or visceral organs (2). Ovarian lymphangiomas, however, are extremely rare, with a limited number of well-documented cases (3, 4). Smaller ovarian lymphangiomas do not exhibit typical clinical symptoms and are often discovered incidentally through imaging studies, whereas larger ones may present with common pelvic symptoms such as abdominal distension and pain (5). Ovarian lymphangiomas usually appear as multilocular cystic structures with fluid content in current imaging modalities, lacking specificity. Due to their rarity and the absence of specific diagnostic criteria, ovarian lymphangiomas are often misdiagnosed as other tumors. Ovarian lymphangiomas are essentially benign, with good prognosis after surgical removal and a low risk of recurrence (3, 4, 6). We reported a case of bilateral ovarian lymphangioma in a postmenopausal female patient, who underwent successful surgery, recovered well postoperatively, and showed no signs of recurrence. We hope this report will provide a reference for future research.

## Case report

**Figure 1:**
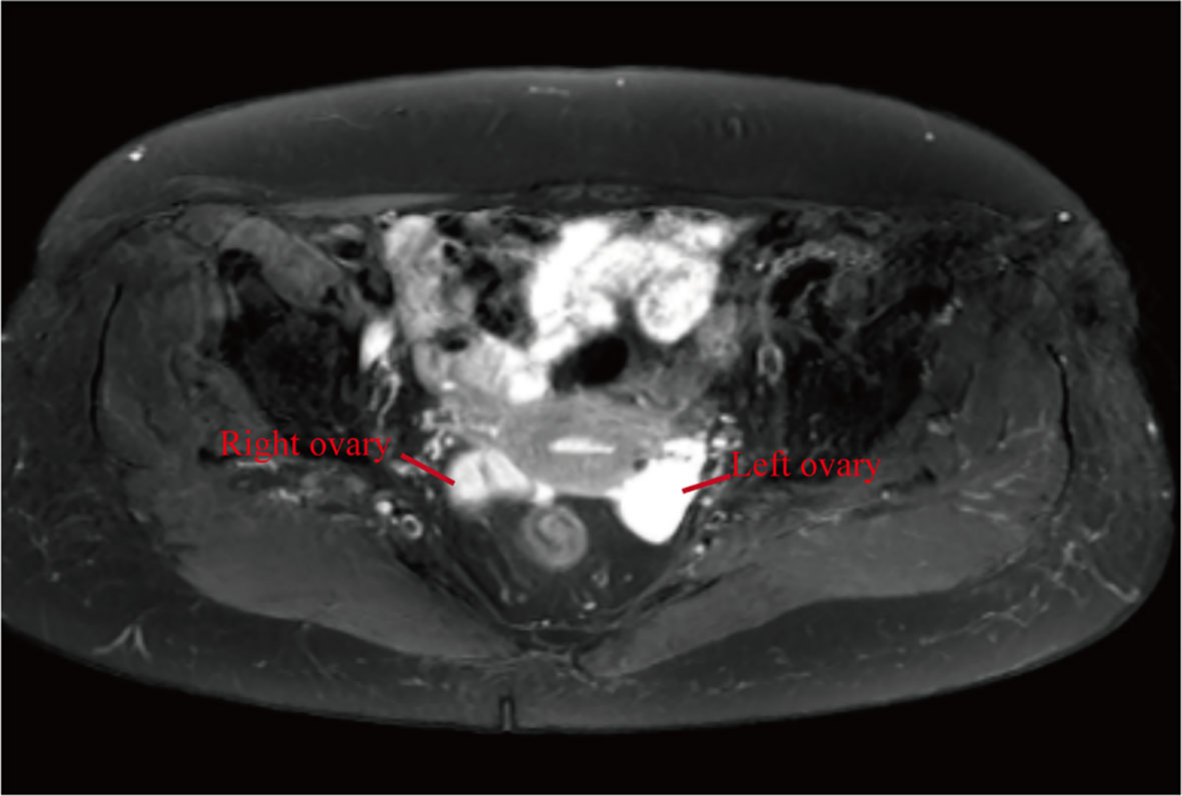
MRI pelvis plain scan and enhancement. Bilateral adnexal area cystic abnormal signal.

**Figure 2:**
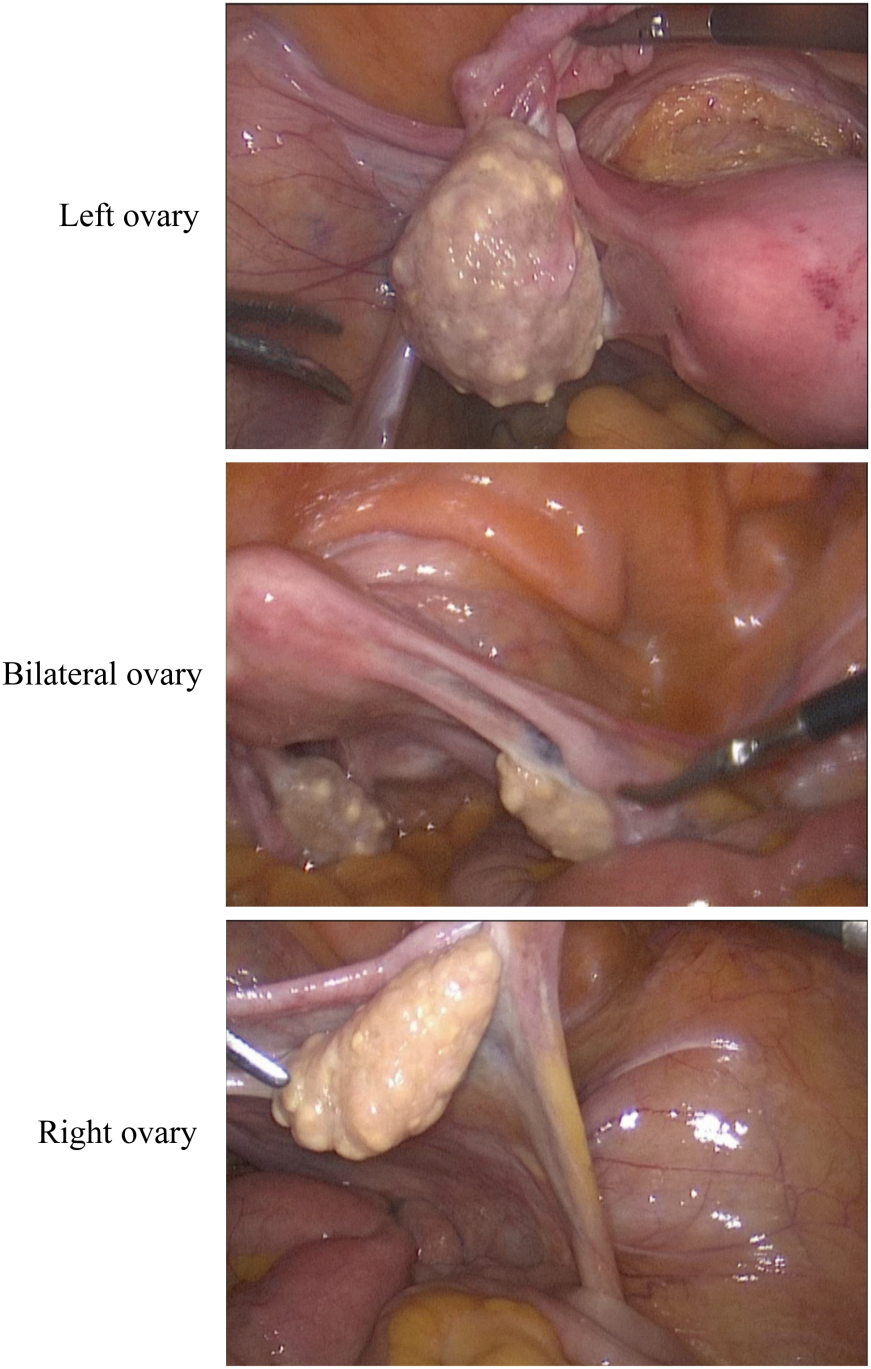
Laparoscopic surgery pictures. The volume of both ovaries increased slightly, and granular changes were observed on the surface.

**Figure 3:**
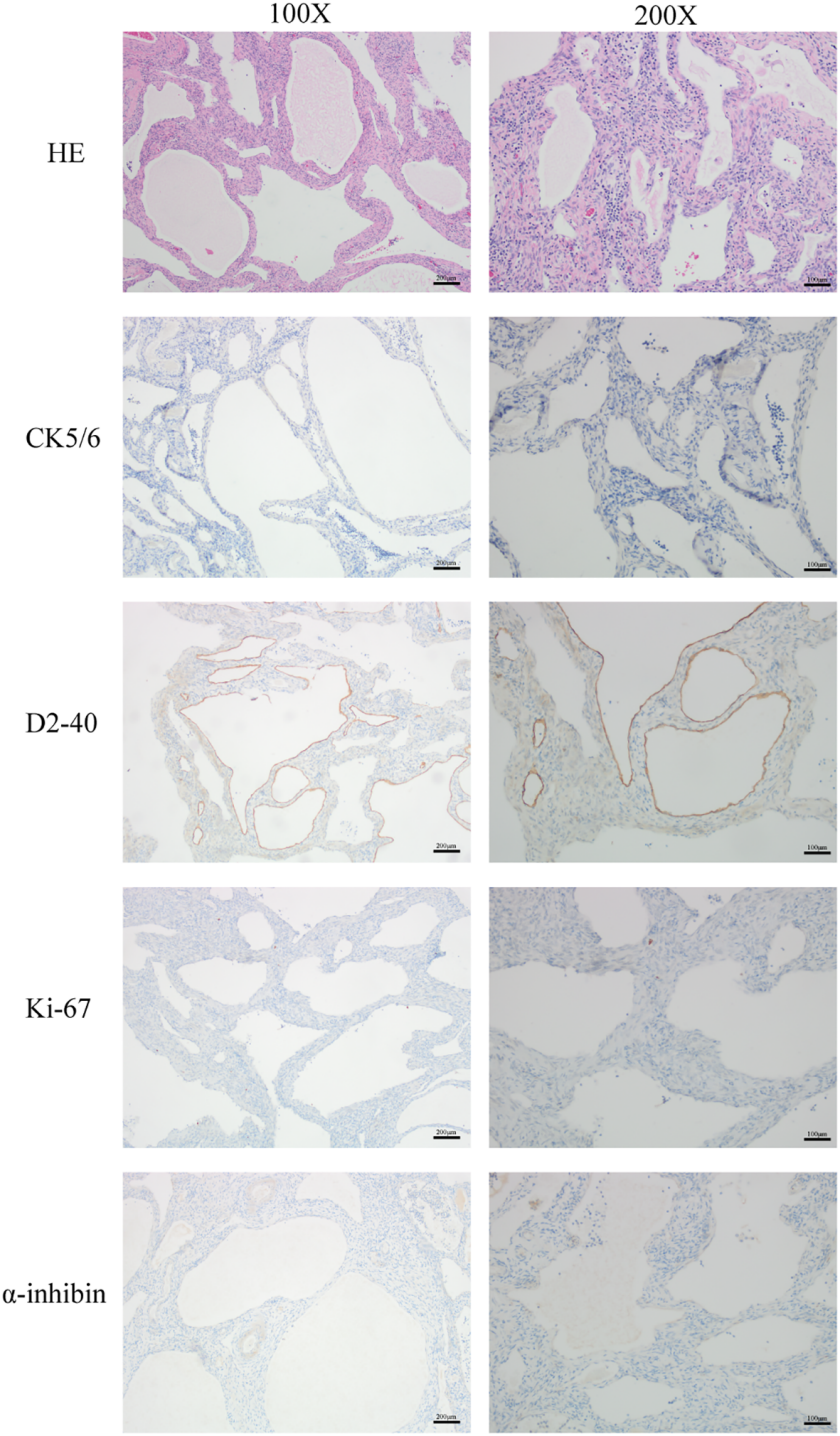
H&E staining was used to show the morphology and structure of the cells in this tumor tissue. The expression of CK5/6, D2-40, Ki67 and α-inhibin were determined by immunohistochemistry. Scale bar: 200 μm, magnification: 100X, Scale bar: 100 μm, magnification: 200X.

1. The patient is a postmenopausal female, aged 69, who presented to our hospital in January 2024 with a complaint of “ovarian tumor discovered over a month ago.” The patient had been menopausal for 24 years, had three children, experienced one abortion, and used tubal ligation for contraception.
2. The patient exhibited no typical clinical symptoms. She had a history of hypertension for more than 10 years, with oral medication to control blood pressure, and a history of atrial fibrillation, which was not treated. She went a bilateral tubal ligation surgery 30 years ago. She had no fear of cold or fever, cough or sputum, abdominal pain, abdominal distention, or irregular vaginal bleeding. No family history of specific diseases. She recently slept well, had a good diet, had normal bowel and urine, and had no significant change in weight. During a routine health check-up in November 2023, abdominal ultrasound revealed mixed masses in both ovaries, with the left measuring approximately 30×31×21mm^3^ and the right measuring 20×16×18mm^3^. Routine hematological and biochemical indicators on admission included complete blood counts and serum electrolytes within normal ranges. Tumor markers like AFP, CEA, CA-125, CA-199 and HE-4 were within normal range.
3. Upon admission, an MRI examination was conducted on the patient, revealing bilateral adnexal area cystic abnormal signal (cystadenoma not excepted) (**Figure 1**), uterine fibroids. Serum tumor markers were within normal ranges, and chest CT and ECG showed no abnormalities. During the gynecological examination, no obvious masses were palpable in the adnexal regions. Preoperative diagnosis was bilateral ovarian tumors and uterine fibroids. Subsequently, the patient underwent surgery, specifically laparoscopic total hysterectomy and bilateral salpingo-oophorectomy. Intraoperatively, both ovaries were noted to be slightly enlarged, approximately 3.5cm in diameter, with granular protrusions on the surface (**Figure 2**), and both fallopian tubes showed changes consistent with post-ligation. There was no pelvic adhesion or fluid, and the upper abdomen was unremarkable. The intraoperative rapid pathology report indicated a multilocular cystic lesion in both ovaries, with some areas lined by flattened epithelium and fibrous collagenous cyst walls, showing relatively mature differentiation. The surgery proceeded smoothly, and postoperative supportive treatments including anti-inflammatory measures were administered. Considering the patient’s good recovery, she was discharged.
4. Postoperative pathology and immunohistochemistry confirmed bilateral ovarian lymphangiomas. As shown in **Figure 3**, lymphangioid structures containing lymph fluid and scattered lymphocytes can be seen in the tumor tissue from the pictures stained by HE. Further immunohistochemistry was performed, and the results suggested: CKpan (-), calretinin (-), CK5/6 (-), D2-40 (+), pax-8 (-), α-inhibin (-), Ki-67 (+, 1%), EMA (-), ER (stromal +). Positive expression of D2-40 can diagnose lymphoma. No expression of CK5/6 and α-inhibin ruled out adenomatoid tumors and ovarian sex cord-stromal tumor (**Figure 3**).
5. The patient underwent surgery in January 2024 and was followed up for more than 8 months. No significant abnormalities were found in examination and gynecological examination.

## Summary

We collected the reports about ovarian lymphangioma in the last 10 years, and summarized the age of the patient, symptoms, size of the tumor, treatment modalities, and recurrence (**Table 1**). Symptoms vary in these cases, including lower abdominal pain and dysfunctional uterine bleeding (3), abdominal painless abdominal distention (4), chylous ascites in pregnancy (7), severe dysmenorrhea, oligomenorrhoea and chyluria (12) and infertility (13). Laparoscopic exploration was performed in only one case, and at least oophorectomy was performed in the rest. In all cases, there was no recurrence during six-month to 2-year follow-up, except for one case there was mild chylous ascites with chyluria at 3 months follow-up (12).

**Table 1:** Related reports of ovarian lymphangioma in recent 10 years.

	Researcher	Age(years)	Symptom	Position and size	Treatment mode	Recurrent situation
1	Sharma A et al.(3)	33	Lower abdominal pain and dysfunctional uterine bleeding	Right ovary, 4x3x2 cm3	Exploratory laparotomy, right ovarian tumor resection and right salpingectomy	There was no recurrence at 6 months follow-up
2	Pani E et al.(4)	16	Abdominal painless abdominal distention	Left ovary, 40x15x29cm3	Left oophorectomy and salpingectomy with the excision of the lesion	There was no recurrence at 2 years follow-up
3	Radhouane A et al.(13)	36	Infertility	Right ovary, no mention	Exploratory laparoscopy	There was no recurrence at 1 years follow-up
4	Choudhary RA et al.(7)	36	Chylous ascites in pregnancy	Bilateral ovary, right ovary-8x7 x5 cm3, left ovary-7x6x5 cm3	Caesarean section and bilateral oophorectomy	There was no recurrence at 6 months follow-up
5	Nerune SM et al.(12)	35	severe dysmenorrhoea, oligomenorrhoea and chyluria	Bilateral ovary, right ovary-8x3x1cm3, left ovary-4x2x1.5 cm3	Total abdominal hysterectomy with bilateral salpingo-oophorectomy	There was mild chylous ascites with chyluria at 3 months follow-up

The histopathological results were similar, sections studied through ovary showed multiple cystic (lymphatic) spaces of varying sizes lined by flattened endothelial cells separated by thin fibrocollagenous septae. In lymphatic spaces numerous lymphocytes was noted in the lumen.

## Discussion

Lymphangioma is a rare benign tumor originating from lymphatic endothelial cells. It typically occurs in children and young adults but can present at any age. Lymphangiomas can develop in any part of the body, but they are most common in the head and neck, particularly the neck and armpits (2). Ovarian lymphangiomas, however, are extremely rare, with reports indicating that retroperitoneal lymphangiomas account for only 1% of cases (6).

There is no clear consensus on the etiology and pathogenesis of ovarian lymphangiomas in existing research. Some studies suggest that abnormal development of the lymphatic system during embryonic growth may be a significant cause of ovarian lymphangiomas, with the lesion progressively developing as the embryo matures (8). However, this viewpoint is not widely accepted. A majority of researchers believe that chronic inflammation and certain infections over an extended period may stimulate lymphatic cell proliferation, and lymphatic obstruction leading to local accumulation of lymph fluid could further induce ovarian lesions, forming ovarian lymphangiomas (9). Pathologists suggest that a more accurate term for this condition might be lymphangiectasia, as the primary cause is likely lymphatic obstruction (10). Some researchers also believe that when ovarian tumor patients undergo radiation therapy, damage to regional lymphatic drainage can lead to the formation of lymphangiomas (11).

Although estrogen is closely associated with the development of some ovarian tumors, there is currently no direct evidence to suggest that estrogen plays a major role in the occurrence of ovarian lymphangiomas. In this case, the patient has no prior history of tumor or chronic lymphangitis, and no clinical symptoms. She has undergone bilateral tubal ligation. We speculate that this might have been due to inadequate lymphatic drainage from the ovaries as a result of the tubal ligation procedure, although there is insufficient evidence to support this hypothesis.

Ovarian lymphangiomas lack specific clinical symptoms. Smaller lymphangiomas do not produce typical abdominal symptoms, while larger ones may cause abdominal compression symptom, pressing on adjacent organs and leading to symptoms such as frequent urination, constipation, and abdominal pain (5). Some reports also indicate that lymphangiomas can be associated with chylous ascites, with the volume of ascites varying with the menstrual cycle, notably increasing during the ovulation period (6). When dealing with women of childbearing age, we should be more vigilant about the formation of ovarian lymphangiomas in cases where ascites symptoms vary in volume with the menstrual cycle. In this case, the patient is a postmenopausal woman with no clinical symptoms, and the condition was only discovered during a routine physical examination. Chylous ascites was not observed during the surgery. The absence of symptoms may be related to the small size of the ovarian lymphangioma.

The diagnosis of ovarian lymphangioma relies on pathological examination. Imaging studies such as ultrasound, CT, and MRI often indicate a cystic ovarian mass, which can be difficult to distinguish from other ovarian tumors. In addition to ovarian tumors, the main differential diagnoses for lymphangiomas include hemangiomas and adenomatoid tumors (5). Ovarian lymphangiomas are composed of dilated lymphatic vessels lined with endothelial cells. Under the microscope, these lymphatic channels contain lymph or chyle. Hemangiomas, on the other hand, are filled with a large number of red blood cells, making them easily distinguishable from lymphangiomas (12). Furthermore, lymphangiomas have a relatively simple cellular composition, primarily consisting of endothelial cells, with a lack of significant cellular atypia and mitotic figures, which helps to differentiate them from malignant tumors. The distinction between lymphangiomas and adenomatoid tumors relies on staining of the pathological sections, including periodic acid-Schiff (PAS) and Alcian blue staining. MRI examination of this case suggests that the cyst should be differentiated from cystadenoma on imaging.

Since they do not naturally regress, surgical treatment is the common approach for ovarian lymphangiomas. The common surgical methods include laparoscopic surgery and open surgery. In previous pathological reports, there have been very few instances of lymphangioma recurrence after surgery (3, 4, 13). In one case report, the patient experienced mild chylous ascites three months after surgery, but this symptom of chylous ascites and chyluria was present before the operation. The report did not mention a recurrence of the lymphangioma (12).

In our case, the patient underwent laparoscopic total hysterectomy and bilateral salpingo-oophorectomy. Follow-up has been conducted for more than eight months post-surgery, and ultrasound examinations have confirmed no signs of recurrence. Given the low rate of postoperative recurrence and the fact that the diagnosis of ovarian lymphangioma relies on surgical pathology, we can consider surgical treatment to be the best current therapeutic approach.

## Conclusion

Ovarian lymphangiomas are quite rare and lack specific diagnostic criteria, yet as clinicians, we must remain highly vigilant for this condition, particularly in patients presenting with unexplained ovarian tumors accompanied by ascites. Surgical treatment is currently the best approach for ovarian lymphangiomas, with a low rate of recurrence post-operatively, and pathological diagnosis remains the gold standard.

## Data Availability

The original contributions presented in the study are included in the article/Supplementary Material. Further inquiries can be directed to the corresponding authors.
